# Preemptive left stellate ganglion block reduces the incidence and severity of cardiac surgery-associated acute kidney injury: a randomized clinical trial

**DOI:** 10.1097/JS9.0000000000002913

**Published:** 2025-07-08

**Authors:** Wei Zhou, Yanlong Yu, Shunping Tian, Hao Wu, Jiajia Yin, Chao Chen, Shantian Feng, Kai Zhang, Rongrong Ma, Zhi Xing, Jianyou Zhang, Zhuan Zhang

**Affiliations:** a Department of Anesthesiology, The Affiliated Hospital of Yangzhou University, Yangzhou University, Yangzhou, Jiangsu, China; b Department of Anesthesiology, Southeast University Affiliated Xuzhou Central Hospital, Southeast University, Xuzhou, Jiangsu, China; c School of Medicine, Yangzhou University, Yangzhou, Jiangsu, China

**Keywords:** acute kidney injury, cardiac surgery, renal artery, resistive index, stellate ganglion block, sympathetic nerve

## Abstract

**Background:**

Acute kidney injury is a common and severe complication of cardiac surgery. A connection might exist between renal sympathetic nerves and left stellate ganglion. It remains unclear whether preemptive left stellate ganglion block (SGB) can effectively prevent cardiac surgery-associated acute kidney injury (CSA-AKI) in clinical practice.

**Method:**

Participants were randomly assigned to SGB group with 0.375% ropivacaine 5 ml performed post-general anesthesia induction or control group (no SGB). The primary outcomes were incidence and severity of CSA-AKI within 7 days postoperatively. Secondary outcomes were intraoperative resistive index (RI) and pulsatility index (PI) of left renal artery via TEE and perioperative IL-6, CRP, and norepinephrine. RR and 95% CI were calculated to compare outcomes between groups. Sensitivity analyses were performed to confirm robustness of findings.

**Result:**

A total of 138 participants were randomized for intention-to-treat (ITT) analysis (69 SGB, 69 control) and 119 for per-protocol (PP) analysis (59 SGB, 60 control). In the ITT analysis, the incidence of CSA-AKI was significantly lower in the SGB group than the control group (14.5% [10/69] vs. 40.6% [28/69], RR 0.351, 95% CI: 0.169–0.728, *P* = 0.005). The PP analyses (13.6% [8/59] vs. 41.7% [25/60], RR 0.325, 95% CI: 0.160–0.660, *P* = 0.001) demonstrated similar results. The severity of CSA-AKI was significantly lower in the SGB group than the control group (ITT and PP: *P* < 0.001). The RI and PI were significantly lower in the SGB group than the control group at post-CPB cessation (*P* < 0.001 and *P* = 0.005, respectively). Postoperatively, the SGB group demonstrated significant reductions in IL-6, CRP, and norepinephrine (all *P* < 0.05). The sensitivity analysis confirmed the robustness of the observed effects, yielding an unadjusted benefit ratio of 0.244 (95% CI: 0.096–0.620, *P* = 0.003) for the incidence of CSA-AKI and 0.197 (95% CI: 0.082–0.468, *P* < 0.001) for its severity.

**Conclusion:**

Preemptive left SGB effectively reduces the incidence and severity of CSA-AKI in patients undergoing cardiac surgery under CPB.

## Introduction

Cardiac surgery-associated acute kidney injury (CSA-AKI) is one of the most common major complications of cardiac surgery, with incidence rates ranging from 5% to 42%[1]. Severe CSA-AKI can increase perioperative mortality rates by 3 to 8 times, prolong the length of stay in the intensive care unit (ICU) and the hospital, and increase healthcare costs[2]. The pathophysiology of CSA-AKI is complex and probably involves multiple factors, including renal hypoperfusion, neurohumoral activation, inflammation and oxidative stress, and nephrotoxic drugs[3]. Efforts to develop strategies for the prevention of CSA-AKI have thus far yielded limited results.

Increased renal sympathetic nerve activity has been shown to induce renal artery vasoconstriction, and cause structural injury in ischemic AKI models[4]. Evidence from Tsai *et al*[5] demonstrated that renal sympathetic denervation significantly reduced the neural activity of the left stellate ganglion. Removal of the renal sympathetic nerves in dogs significantly reduced the occurrence of atrial fibrillation induced by left stellate ganglion stimulation, suggesting a potential functional interplay between renal sympathetic nerves and the left stellate ganglion[6]. Based on these findings, we hypothesize that pretreatment with left stellate ganglion block (SGB) prior to cardiac surgery may enhance renal blood flow (RBF) and reduce the incidence and severity of CSA-AKI. Real-time monitoring of left RBF using transesophageal echocardiography (TEE) could provide valuable insights into intraoperative renal perfusion dynamics, enabling early detection and intervention in cases of impaired renal hemodynamics.

To test our hypotheses, the primary objective of this prospective, randomized, controlled trial was to evaluate the effect of preemptive left SGB on the incidence and severity of CSA-AKI in patients undergoing cardiac surgery with cardiopulmonary bypass (CPB). The secondary aim was to explore the intraoperative changes in RBF by using TEE. There were no uses of artificial intelligence throughout the whole manuscript, and it complies with the TITAN 2025 guidelines[7].

## Methods

### Study design

The study was approved by the ethics committee of the Affiliated Hospital of Yangzhou University (2023-YKL01-Project 9) and registered on the ClinicalTrials.gov website (NCT05652179) on 7 November 2022. Informed consent was obtained from all the participants. This clinical trial adheres to the applicable CONSORT guidelines[8]. This study is based on the work of our master’s thesis and shares the same research plan, sample frame and basic data collection.

### Participants

Participants who were scheduled to undergo cardiac surgery via a midline thoracic incision under CPB were recruited between January 2023 and December 2023. Adult patients of any gender, with American Society of Anesthesiologists (ASA) class of III or IV, were eligible. The first participant was enrolled on 10 January 2023. The exclusion criteria included emergency cardiac surgery, major vascular surgery, non-sinus rhythm, reoperation, contraindications for TEE or SGB, abnormal preoperative renal function (creatinine >133 μM[9] or chronic kidney disease history), severe preoperative heart failure with left ventricular ejection fraction <30%, multi-organ dysfunction, enrolled in another clinical trial, and severe infection requiring continuous antibiotic treatment.

During the registration of this trial, the ASA class inclusion criteria were mistakenly marked as II or III (the actual implementation standard is III or IV). All enrolled patients strictly met the ASA class III or IV criteria. This error is limited to the registration platform information and does not affect the actual trial design and patient screening process. Although there is a delay in correcting the ASA classification standards in the registration information, the actual implementation standards are completely consistent with the research protocol.

### Randomization and blinding

Before entering the operating room, the enrolled participants were divided into the following 2 groups at a 1:1 ratio by using a computer-generated random number table: the SGB group (0.375% ropivacaine 5 ml injected around the stellate ganglion) and the control group (no SGB). All the participants were blinded to the group allocation. The group assignments were concealed in sealed envelopes, which were handed to the anesthesiologist who perform the SGB; this anesthesiologist was not involved in the implementation of the anesthesia protocol and data collection in this study. TEE examination, which was synchronized with ECG monitoring, was performed by 2 anesthesiologists who were unaware of the grouping criteria, and both of whom had more than 5 years of TEE experience. All the result parameters were recorded by an anesthesiologist who was unaware of the group assignments, and was not involved in the implementation of the anesthesia protocol.

**HIGHLIGHTS**

- This study aimed to evaluate the effect of preemptive left stellate ganglion block (SGB) on cardiac surgery-associated acute kidney injury (CSA-AKI).
- Left SGB decreases both the incidence and severity of CSA-AKI in patients undergoing cardiac surgery under cardiopulmonary bypass.
- Renal arterial perfusion detected under transesophageal echocardiography was significantly improved intraoperatively.

### Protocol

**Figure 1.**
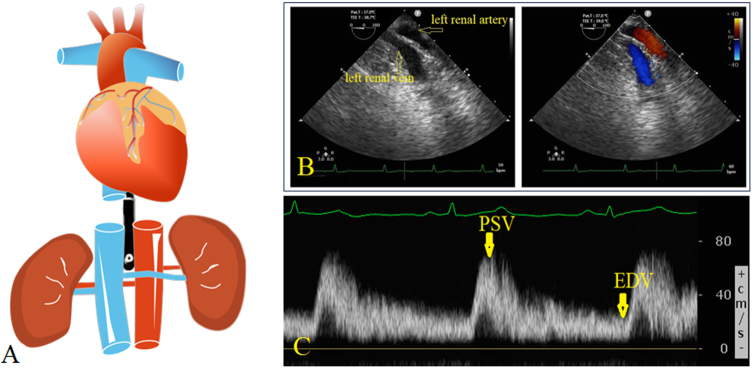
Left renal artery blood flow examination under TEE. A, schematic diagram of the procedure for detecting the left renal artery blood flow by TEE; B, the nearly parallel left renal artery and vein under TEE (without and with blood flow color indicated); C, renal arterial blood flow velocity pattern recorded with pulsed-wave Doppler tracing, in which the highest point of the waveform is peak systolic velocity (PSV) and the trough is the end-diastolic velocity (EDV), and tracing the Doppler flow curves helps obtain the mean velocity.

The TEE probe (X7-2 t) of the color Doppler ultrasound system (Philips CX50 Color Doppler Ultrasound Diagnostic System, Royal Philips, Amsterdam, The Netherlands) was inserted into the esophagus after induction of general anesthesia. The TEE results were confirmed after consultation between the 2 anesthesiologists. The first TEE examination was implemented to acquire the baseline values of left RBF parameters and to perform a rapid assessment of cardiac function. For examination of RBF parameters using TEE, the probe was advanced from the upper incisors to obtain a standard mid-esophageal 4-chamber view, and then further advanced to obtain a transgastric short-axis view. The probe was then rotated 90°–270° to the left until the short axis of the descending aorta appeared in the center of the field of view. The oval hypoechoic area behind the descending aorta corresponds to the vertebral body. The probe was then advanced gently and smoothly until the nearly parallel left renal artery and vein appeared in the field of view (Fig. 1). Following the left renal artery, the probe could be turned right about 90° to visualize the left kidney. The TEE anesthesiologists were asked to be absent for 20 min after the first TEE examination was completed.

In the SGB group, ultrasound-guided ropivacaine injection around the left stellate ganglion was performed after the first TEE examination and before surgery initiation. The participant’s head was tilted to the right. A high-frequency probe (6–13 MHz) was placed between the C6 and C7 transverse processes to obtain the best image of the longus colli muscle. After iodine disinfection, a 22-G atraumatic needle for peripheral nerve blocks (B. Braun Melsungen AG, Melsungen, Germany) was used to puncture the site posterior to the left carotid artery and on the surface of the longus colli muscle via an in-plane technique. Then, 5 ml of 0.375% ropivacaine hydrochloride injection was administered after confirming negative aspiration for blood, cerebrospinal fluid, or air. Successful blockade was indicated by increased skin temperature on the left side of the head, face, and upper limb; and increased perfusion index on left pulse oximetry[10]. Any complications (e.g., hypotension, arrhythmias, and local redness or swelling) were recorded. Participants underwent a second TEE examination for the assessment of left RBF parameters and a thorough evaluation of heart disease and cardiac function at 15 min after SGB implementation in the SGB group. Usually, the SGB procedure could be completed within 5 min. Therefore, in the control group, a second TEE examination was implemented at 20 min after the completion of the first TEE examination.

All surgeries were performed by the same proficient team of cardiac surgeons. Standard anesthesia care and CPB protocol of our institution were implemented for all patients. All participants were transferred to the ICU postoperatively for standard institutional care.

### Outcome variables

The primary outcomes were the incidence and severity of CSA-AKI in the 7 postoperative days. CSA-AKI was diagnosed using the Kidney Disease: Improving Global Outcomes (KDIGO) criteria[11]. The severity of CSA-AKI was graded by the change in plasma creatinine levels, according to the KDIGO criteria: stage 1, increase in serum creatinine by ≥0.3 mg/dL (≥26.5 µmol/L) within 48 h after cardiac surgery, or increase in serum creatinine to 1.5–1.9 times the baseline level; stage 2, increase in serum creatinine to 2.0–2.9 times the baseline level; and stage 3, increase in serum creatinine to 3.0 times the baseline level, or increase in serum creatinine to ≥ 4.0 mg/dL (≥353.6 µmol/L), or initiation of continuous renal replacement therapy (CRRT). The baseline reference for serum creatinine was obtained on the last weekday before the surgery[12]. Serum creatinine was measured upon surgery completion and once a day from the patient’s arrival in the ICU until postoperative day 7 to diagnose the incidence and severity of CSA-AKI. Urine volume was not considered for CSA-AKI diagnosis due to the use of hemofiltration during CPB and the administration of diuretics in most patients during the perioperative period.

Secondary outcomes included the resistive index (RI) and pulsatility index (PI) of the left renal artery, which were measured after induction of general anesthesia (baseline, post-GA induction), at the second TEE examination, and 30 min after CPB cessation (post-CPB cessation). The RI was calculated as (PSV − EDV)/PSV, and PI as (PSV − EDV)/(V-mean), in which PSV is peak systolic velocity, EDV is end-diastolic velocity, and V-mean is mean blood flow velocity. Each indicator was measured over 3 consecutive cardiac cycles at each time point, and the average value was calculated. Venous blood samples were also collected to test interleukin (IL)-6, C-reactive protein (CRP), norepinephrine, and creatinine levels on the last weekday before the surgery (baseline), upon surgery completion, at 24 h postoperatively, 48 h postoperatively, and 7 d postoperatively. The urine output was recorded on the first and second postoperative days.

Safety outcomes included the duration of postoperative mechanical ventilation in ICU, length of ICU stay, length of postoperative hospitalization in the ward, use of CRRT within 30 days after the surgery, and in-hospital mortality.

We also recorded the patients’ demographic data, including sex, age, height, weight, body mass index, ASA classification, preoperative cardiac function classification, and history of hypertension and diabetes, as well as surgery-related information, including surgery type, anesthesia duration, surgery duration, CPB duration, aortic cross-clamp duration, blood loss, transfusion rate, intraoperative fluid infusion, intraoperative urine output (before, during, and after CPB), ultrafiltration during CPB, and anesthetics and vasoactive medications used during surgery. The incidence of SGB complications, including hypotension, arrhythmias, and local redness or swelling, was also recorded.

### Sample size calculation

Sample-size calculation was performed using PASS (version 15.0) software. The main primary outcome measure was the incidence of CSA-AKI. In our preliminary trial (unpublished), the incidence of CSA-AKI in the SGB group and the control group was 14.3% (3/21) and 40.9% (9/22), respectively. A two-sided test with α = 0.05 and power (1 − β) of 0.9 was conducted. The results showed that each group required a minimum of 55 participants. Considering a dropout rate of 20%, we recruited at least 69 patients in each group.

### Statistical analysis

Data analysis was performed using SPSS statistical software version 25.0 (IBM SPSS Statistics, IBM Corp., Armonk, NY) and SAS 9.4 (SAS Institute Inc., Cary, NC). The Kolmogorov-Smirnov test was used to assess normality. Normally distributed continuous variables were presented as mean (standard deviation) and nonnormally distributed variables were presented as median (IQR). The independent sample t test was used for normally distributed data, and the Mann–Whitney U test was used otherwise for nonnormally distributed data. Differences in repeated-measures variables were analyzed by repeated-measures analysis of variance. Categorical variables were presented as numbers (percentages), and compared using the chi-square test or Fisher exact test.

We did the primary analysis according to the intention-to-treat (ITT) principle (i.e., included all participants as if they were treated according to the group into which they were assigned), and the per-protocol (PP) analyses (i.e., including all participants according to the treatment they actually received). Secondary outcomes were analyzed in the ITT population analysis. The safety outcomes were analyzed in the PP analysis.

To handle missing data, multiple imputations by chained equations were used, assuming missing data at random. To address dropouts and missing data, we used multiple imputation with the Fully Conditional Specification (FCS) method, generating 10 imputed datasets. Results from each dataset were pooled using Rubin’s rules leading to more accurate and robust statistical inferences. For secondary outcomes, a Benjamini–Hochberg correction was used to control the false discovery rate, ensuring the validity of statistical inferences. Separate corrections were performed for the ITT and PP analyses due to differences in the number of secondary outcomes considered in each population.

We did a sensitivity analysis for the primary outcomes using a multivariable logistic regression model, with severity assessed using an ordinal logistic regression approach. The model included covariates such as sex, age, BMI, ASA physical status, NYHA classification, hypertension, diabetes, surgery type, CPB duration, and aortic cross-clamp duration. To facilitate the calculation and interpretation of odds ratios, continuous variables were categorized into binary groups based on clinically relevant or commonly used thresholds.

Analysis items with 2-sided *P* <0.05 were considered statistically significant. Specifically, we applied a two-sided significance level of 0.025 (α = 0.05/2) to both primary outcome analyses due to the Bonferroni correction applied.

## Results

### General characteristics of the participants

After subsequent verification, the ASA class of all participants included in the analysis was confirmed by two senior chief anesthesiologists (Z.Z. and J.Z.) to meet the grade III or IV standard, and the original medical records were complete and traceable.

**Figure 2.**
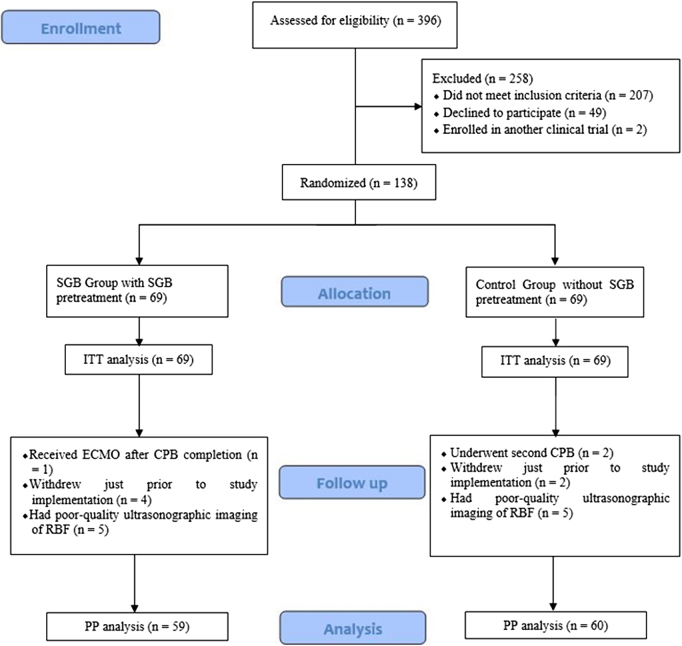
Flow diagram of the trial protocol. CPB, cardiopulmonary bypass; ECMO, extracorporeal membrane oxygenation; ITT, intention-to-treat; PP, per-protocol; RBF, renal blood flow; SGB, stellate ganglion block.

Between January 2023 and December 2023, 396 participants who were scheduled to undergo cardiac surgery were screened for enrollment. Of those, a total of 138 participants were randomized into this study and available for the ITT analysis, with 69 in the SGB group and in the control group each. Ultimately, a total of 119 participants were available for the PP analysis, with 59 in the SGB group and 60 in the control group (Fig. 2).

No statistically significant differences were found between the 2 groups in terms of demographic parameters, surgery-related information, and intraoperative medication usage (*P* > 0.05 for all; Table 1).

**Table 1:** Baseline characteristics and intraoperative data

Parameter	SGB group (n = 69)	Control group (n = 69)	*P*
Sex, n (%)			
Male	44 (63.8)	49 (71)	0.364
Female	25 (36.2)	20 (29)	
Age (yr)	64.1 (9.2)	64.6 (8.7)	0.761
Height (m)	1.6 (0.1)	1.66 (0.1)	0.259
Weight (kg)	65.1 (10.1)	65.21 (11)	0.958
BMI (kg/m^2^)	24.2 (3.1)	23.8 (3.3)	0.434
ASA physical status			
III	6 (8.7%)	7 (10.1%)	0.771
IV	63 (91.3%)	62 (89.9%)	
NYHA classification			
II	10 (14.5%)	10 (14.5%)	1.000
III	59 (85.5%)	59 (85.5%)	
Previous medical history			
Hypertension	41 (59.4%)	43 (62.3%)	0.727
Diabetes, n (%)	20 (29%)	24 (34.8%)	0.465
Stroke, n (%)	6 (8.7%)	5 (7.2%)	0.753
Surgery type			
CABG	44 (63.8%)	44 (63.8%)	0.832
Valve replacement	18 (26.1%)	16 (23.2%)	
CABG + valve replacement	7 (10.1%)	9 (13.0%)	
Anesthesia duration (min)	355.5 (67.3)	366.1 (69.1)	0.362
Surgery duration (min)	301 (65.2)	308.1 (70.8)	0.537
CPB duration (min)	123.5 (33.4)	129.48 (39.1)	0.337
Aortic cross-clamp duration (min)	92.5 (26.3)	97.9 (34.3)	0.302
Blood loss (mL)	300 (275, 360)	300 (300, 370)	0.591
Transfusion	37 (53.6%)	35 (50.7%)	0.733
Intraoperative fluid infusion (mL)	5258.8 (846.5)	5206.01 (836.5)	0.713
Intraoperative urine output (mL)			
Before CPB	150 (80, 220)	100 (50, 200)	0.178
During CPB	300 (200, 500)	290 (180, 500)	0.441
After CPB	400 (300, 500)	300 (200, 500)	0.052
Ultrafiltration during CPB (mL)	2500 (1650, 3500)	2800 (1900, 3500)	0.606
Intraoperative dosages of anesthetics and vasoactive medications			
Propofol (mg · kg^-1^ · h^-1^)	5 (1.4)	5.1 (2.1)	0.684
Sufentanil (μg/kg)	4 (1.2)	3.6 (0.9)	0.061
Remifentanil (μg · kg^-1^ · min^-1^)	0.1 (0.1)	0.1 (0.1)	0.865
Epinephrine (μg/kg)	2.3 (2.7)	2.9 (2.3)	0.162
Norepinephrine (μg/kg)	4.8 (3.9)	5 (3.5)	0.802
Nitroglycerin (μg/kg)	63 (49.7)	59 (46.7)	0.628
Creatinine at baseline (μmol/L)	71.9 (16)	70.9 (15.1)	0.743
IL-6 at baseline (ng/L)	5.2 (3.2)	5.2 (2.9)	0.978
CRP at baseline (mg/L)	2.9 (2.8)	3.4 (4)	0.420
Norepinephrine at baseline (pg/mL)	316 (109.2)	329.5 (115.5)	0.506
RI at post-GA induction	0.7 (0.1)	0.7 (0.1)	0.734
PI at post-GA induction	1.4 (0.3)	1.4 (0.4)	0.881

Data are presented as n (%), mean (SD), or median (IQR).

ASA, American Society of Anesthesiologists; BMI, body mass index; CABG, coronary artery bypass grafting; CPB, cardiopulmonary bypass; CRP, C-reactive protein; ICU, intensive care unit; IQR, interquartile range; NYHA, New York Heart Association; PI, pulsatility index; RI, resistive index; SD, standard deviation; SGB, stellate ganglion block.

### Primary outcomes

In the ITT analysis, the incidence of CSA-AKI was significantly lower in the SGB group than in the control group (14.5% [10/69] vs. 40.6% [28/69], RR 0.351, 95% CI: 0.169–0.728, *P* = 0.005). The PP analyses (13.6% [8/59] vs. 41.7% [25/60], RR 0.325, 95% CI: 0.160–0.660, *P* = 0.001) demonstrated similar results as the ITT analysis. In the ITT analysis, 13 (18.84%) patients were diagnosed with CSA-AKI in stage 1, 9 (13.04%) patients with CSA-AKI in stage 2, and 7 (10.14%) patients with CSA-AKI in stage 3 in the control group; 8 (11.59%) patients developed CSA-AKI in stage 1, and 2 (2.90%) patient developed CSA-AKI in stage 2 in the SGB group. In the PP analysis, 11 (18.3%) patients were diagnosed with CSA-AKI in stage 1, 8 (13.3%) patients with CSA-AKI in stage 2, and 6 (10%) patients with CSA-AKI in stage 3 in the control group; 7 (12.5%) patients developed CSA-AKI in stage 1, and 1 (1.7%) patient developed CSA-AKI in stage 2 in the SGB group. The severity of CSA-AKI was significantly lower in the SGB group than in the control group (ITT and PP: *P* < 0.001; Table 2).

**Table 2:** Incidence and severity of cardiac surgery-associate acute kidney injury

	SGB group	Control group	RR/OR	95% CI	*P*
**Incidence**					
ITT	10 (14.5%)	28 (40.6%)	0.351	0.169–0.728	0.005
PP	8 (13.6%)	25 (41.7%)	0.325	0.160–0.660	0.001
**Severity**					
ITT			0.197	0.082–0.468	<0.001
Stage 1	8 (11.6%)	13 (18.8%)			
Stage 2	2 (2.9%)	9 (13.0%)			
Stage 3	0 (0.00%)	7 (10.1%)			
PP			0.198	0.080–0.489	<0.001
Stage 1	7 (12.5%)	11 (18.3%)			
Stage 2	1 (1.7%)	8 (13.3%)			
Stage 3	0 (0.00%)	6 (10.0%)			

Data are presented as n (%). For ITT analysis, n =69 in each group; for PP analysis, n =59 in SGB group and n =60 in the control group.

For incidence cases, values represent the relative risk (RR) (95% CI); for severity cases, values represent the odds ratio (OR) (95% CI).

A Bonferroni correction with a two-sided significance level of 0.025 (α = 0.05/2) was applied.

ITT, intention-to-treat; OR, odds ratio; PP, per-protocol; RR, relative risk; SGB, stellate ganglion block.

### Secondary outcomes

The RI of the left renal artery was significantly lower in the SGB group than in the control group at the second TEE examination (*P* < 0.001) and post-CPB cessation (*P* < 0.001). The PI of the left renal artery did not differ between the two groups at the second TEE examination (*P* = 0.247), while it was significantly lower at post-CPB cessation in the SGB group than in the control group (*P* = 0.005; Table 3).

**Table 3:** Secondary outcomes of the two groups

Parameter	SGB group (n = 69)	Control group (n = 69)	*P* (ITT)
RI			
At the second TEE examination	0.66 (0.09)	0.73 (0.08)	<0.001
At post-CPB cessation	0.69 (0.05)	0.73 (0.06)	<0.001
PI			
At the second TEE examination	1.29 (0.37)	1.42 (0.36)	0.247
At post-CPB cessation	1.370 (0.274)	1.526 (0.286)	0.005
IL-6 (ng/L)			
Upon surgery completion	75.87 (53.21)	180.45 (107.36)	<0.001
24 h postoperatively	174.99 (110.44)	296.08 (160.90)	<0.001
48 h postoperatively	106.06 (98.16)	233.24 (144.98)	<0.001
7 d postoperatively	25.92 (30.68)	61.77 (58.11)	<0.001
CRP (mg/L)			
Upon surgery completion	15.42 (19.47)	32.29 (35.61)	<0.001
24 h postoperatively	74.10 (36.07)	102.73 (64.09)	0.003
48 h postoperatively	90.50 (50.38)	114.18 (66.82)	0.039
7 d postoperatively	31.72 (22.00)	44.62 (40.05)	0.013
Norepinephrine (pg/mL)			
Upon surgery completion	295.71 (92.86)	399.36 (235.10)	0.003
24 h postoperatively	218.57 (63.92)	339.12 (220.53)	<0.001
48 h postoperatively	232.02 (127.69)	328.37 (172.91)	0.001
7 d postoperatively	200.47 (116.28)	225.70 (191.31)	0.357
Creatinine (μmol/L)			
upon surgery completion	72.75 (16.65)	74.73 (21.61)	0.668
24 h postoperatively	83.95 (20.41)	94.48 (27.62)	0.044
48 h postoperatively	80.69 (21.71)	103.97 (33.61)	0.008
7 d postoperatively	68.33 (14.78)	87.31 (42.64)	0.003
Urine output (ml · kg^-1^ · h^-1^)			
1st 24 h postoperatively	1.47 (0.38)	1.14 (0.48)	<0.001
2nd 24 h postoperatively	1.41 (0.40)	1.18 (0.36)	<0.001

Data are presented as mean (SD).

*P* (ITT), intention-to-treat *P* values, calculated by including all randomized participants.

Missing data for participants who dropped out were imputed to maintain the full sample size for analysis.

Outcome is significant after applying Benjamini–Hochberg (BH) correction.

CRP, C-reactive protein; PI, pulsatility index; RI, resistive index; SGB, stellate ganglion block.

Compared with the control group, the SGB group had significantly lower IL-6 levels upon surgery completion, 24 h postoperatively, 48 h postoperatively, and 7 d postoperatively (*P* < 0.001 for all); significantly lower CRP levels upon surgery completion (*P* < 0.001), 24 h postoperatively (*P* = 0.003), 48 h postoperatively (*P* = 0.039), and 7 d postoperatively (*P* = 0.013); and significantly lower norepinephrine levels upon surgery completion (*P* = 0.003), 24 h postoperatively (*P* < 0.001), and 48 h postoperatively (*P* = 0.001). The study groups did not show significant differences in norepinephrine levels at 7 d postoperatively (*P* = 0.357). The creatinine levels were significantly lower in the SGB group than in the control group at 24 h postoperatively (*P* = 0.044), 48 h postoperatively (*P* = 0.008), and 7 d postoperatively (*P* = 0.003). Postoperative urine output was significantly higher in the SGB group than in the control group during the first 24 h after the surgery (*P* < 0.001) and between 24 h and 48 h after surgery (*P* < 0.001; Table 3).

### Safety outcomes

Compared with the control group, participants in the SGB group underwent significantly earlier postoperative extubation (16 h vs. 19 h; MD −3, 95% CI: −5 to 2, *P* < 0.001) and had significantly shorter length of stay in the ICU (44 h vs. 46.0 h, MD −3, 95% CI: −6 to 1, *P* = 0.006) and length of postoperative hospitalization (12 d vs. 14.0 d, MD −2, 95% CI: −3 to 1, *P* = 0.001). Three patients in the control group required postoperative CRRT, while no patients required this treatment in the SGB group. No deaths in hospital occurred in either group. No SGB-related complications were observed in the SGB group (Table 4).

**Table 4:** Safety outcomes of the two groups

	SGB group (n = 59)	Control group (n = 60)	OR/MD (95% CI)	*P*
Postoperative extubation time (h)	16 (15, 18)	19 (18, 25)	−3 (−5 to 2)	<0.001
Length of postoperative ICU stay (h)	44 (42, 47)	46 (43, 68)	−3 (−6 to 1)	0.006
Length of postoperative hospitalization (d)	12 (9, 15)	14 (12, 17)	−2 (−3 to 1)	0.001
Postoperative CRRT	0 (0%)	3 (5%)	7.244 (0.357–147.045)	0.197
Death in hospital	0 (0%)	0 (0%)	NA	>0.999

Data are presented as n (%) or median (IQR) in the second and third columns.

The fourth column shows between-group comparisons.

For variables presented as n (%), the comparison is expressed as odds ratio (OR) with 95% CI.

For variables presented as median (IQR), the comparison is expressed as median difference (MD) with 95% CI.

CRRT, continuous renal replacement therapy; SGB, stellate ganglion block.

### Sensitivity analysis

The sensitivity analysis showed that the odds ratio for the incidence and severity of CSA-AKI remained largely consistent when the variables, including sex, age, BMI, ASA physical status, NYHA classification, hypertension, diabetes, surgery type, CPB duration, and aortic cross-clamp duration were analyzed. It suggests that the observed effect of left SGB on the incidence of CSA-AKI is robust, where the odds ratio without adjustment was 0.244 (95% CI: 0.096–0.620, *P* = 0.0034); the effect of left SGB on the severity of CSA-AKI is also robust, where the odds ratio without adjustment was 0.197 (95% CI: 0.082–0.468, *P* < 0.001) (Supplementary Digital Content 1, available at: http://links.lww.com/JS9/E623).

## Discussion

If not diagnosed and treated promptly, CSA-AKI can prolong ICU stay and overall hospital stay, increase the incidence of perioperative complications and mortality rates, and increase the risk of chronic kidney disease^[13,14]^. Stress during surgery leads to activation of two major neuroendocrine systems, including the hypothalamic-pituitary axis (HPA) and the sympathetic nervous system (SNS)[15]. Excessive neuroendocrine stress response during cardiac surgery is a key pathophysiological mechanism of CSA-AKI. The activated renal SNS and its consequent effects on norepinephrine overflow from nerve endings may trigger tubular cell death, renal inflammation, and fibrogenesis, and are considered to be involved in the development of AKI^[16,17]^. In clinical trials, dexmedetomidine, a highly selective α_2_-adrenergic receptor agonist, has been shown to effectively mitigate perioperative renal injury through its anti-sympathetic effects^[18,19]^. Animal studies have further demonstrated that renal denervation or ganglion blockade can ameliorate ischemic acute kidney failure by inhibiting sympathetic nerve activity^[17,20]^. Based on current evidence, we propose that inhibiting sympathetic nervous activity during the perioperative period may contribute positively to postoperative renal function. Recent studies have demonstrated that stellate ganglion block offers significant benefits for cardiac function^[21,22]^, suggesting its potential to improve renal outcomes in cardiac surgery merits in-depth investigation.

Stellate ganglion block (SGB) involves the injection of a local anesthetic around the stellate ganglion to temporarily and reversibly block both the preganglionic and postganglionic fibers of the stellate ganglion and the SNS. The stellate ganglion serves as a critical node within the sympathetic chain, receiving preganglionic sympathetic fibers that originate from the thoracic spinal cord segments (T1-T10)[23]. Extensive communication pathways link the renal sympathetic nerves to the stellate ganglion, with both preganglionic and postganglionic sympathetic nerve fibers capable of innervating the renal vasculature[5]. Signals from the renal sympathetic nerves that enter the brainstem can influence the neural activity of the left stellate ganglion through the modulation of central sympathetic outflow pathways[24]. This interconnection underscores that the stellate ganglion and the kidney are closely linked rather than functioning independently of one another. In the present study, left SGB pretreatment markedly reduced the incidence and severity of CSA-AKI, which also led to an improvement in postoperative renal function. Ultrasound-guided SGB enhances the safety of the procedure through real-time visualization of tissue structures, thereby reducing the risk of vascular and soft tissue injury. These findings align with our prior research demonstrating the safety and efficacy of ultrasound-guided SGB[25].

We did not perform sham procedure in the control group. The following reasons are considered. All patients in this study received SGB after the induction of general anesthesia. With sedation and analgesia, the pain and stress response induced by the SGB procedure itself were minimal, and its impact on patients was negligible. The SGB injection volume was 5 ml, and its impact on anatomical structures was significantly less than that of fascial plane block. It is worth noting that injecting saline around the stellate ganglion might also have certain sympathetic blocking effects[26]. This decision not to perform sham procedure in the control group aligns with the methodology employed in previous study[27].

The left stellate ganglion contributes more sympathetic tone to the myocardium than the right[21]. In studies focused on the relationship between stellate ganglion and the heart, researchers usually preferred the left side to perform SGB[28]. Moreover, right internal jugular vein puncture is usually performed in cardiac surgeries for fluid and medication infusion. Therefore, we chose the left side to perform SGB in the current study.

The optimal dose and concentration of local anesthetic for SGB vary among different reports. Compared to lidocaine and bupivacaine, ropivacaine offers longer duration and lower cardiac toxicity, making it a more suitable agent for SGB in patients undergoing cardiac surgery. Administration of 5–10 ml of local anesthetic at the C5-C7 levels is generally recommended[29]. Studies have demonstrated that ultrasound-guided injection of 5 ml of solution at the C6 level provides reliable spread from C4 to T1 and adequately reaches the stellate ganglion[30]. Ultrasound guidance also significantly reduces the volume of local anesthetic required for effective nerve blockade. Based on our department protocol for nerve blocks and preliminary trials, the concentration of ropivacaine was set at 0.375% in the current study.

Impaired RBF self-regulation during anesthesia and surgery highlights the importance of real-time intraoperative monitoring and regulation of RBF in high-risk patients[31]. Although postoperative evaluation of RBF using abdominal ultrasonography can predict and access CSA-AKI[32], this method is challenging to implement during cardiac procedures. Studies have indicated that renal artery blood flow can be measured using TEE, which has demonstrated good consistency with abdominal ultrasonography[33]. We applied real-time monitoring of renal artery blood flow through TEE to reflect intraoperative changes in renal perfusion. The renal artery RI, which reflects renal vascular resistance, describes the difference between the PSV and EDV as a fraction of the PSV, and can be used to predict the prognosis of renal-related treatments[34]. RI serves as a reliable predictor for AKI following major surgical procedures, demonstrating strong predictive capability in CSA-AKI prediction[35]. A RI value of 0.70 is generally considered the upper limit of normal in adults, with values above this threshold indicating increased renal vascular resistance[36]. Our findings indicated that the intraoperative RI was significantly lower in the SGB group compared to the control group, suggesting that left SGB can reduce renal vascular resistance. The PI of the renal artery is a measurement of the variability of blood flow velocity within the vessel, and reflects renal perfusion. Studies have shown that an increased PI is closely related to the occurrence of acute or chronic kidney disease[37]. In our study, the intraoperative PI was significantly lower in the SGB group than in the control group, which also indicates that left SGB pretreatment could significantly improve renal perfusion.

The pathophysiology of AKI involves a cascade of inflammation and fibrosis mediated by sympathetic nerve-derived norepinephrine secondary to injury. Renal impairment, leading to a reduction in glomerular filtration rate, impairs the body’s ability to clear inflammatory markers such as IL-6 and IL-β[38]. In this study, the SGB group demonstrated significantly lower levels of pro-inflammatory cytokines, including IL-6 and CRP, compared to the control group at the completion of surgery, as well as at 24 hours, 48 hours, and 7 days postoperatively. Additionally, the SGB group exhibited significantly reduced norepinephrine levels relative to the control group upon surgery completion, at 24 hours and 48 hours postoperatively. Furthermore, we administered SGB prior to the initiation of surgery to optimize its protective effects before CPB.

### Limitations

First, it is a small sample, single-center study, and further large-sample, multi-center trials are necessary to fully investigate the impact of preemptive left SGB on CSA-AKI.

Second, imaging of the right kidney is challenging due to its lower position in the abdominal cavity and its longer distance from the esophagus. In this study, we focused solely on left renal arterial as measured by TEE. We did not perform postoperative abdominal renal Doppler ultrasound analysis, which limited our ability to observe changes in renal arterial parameters at various postoperative time points.

Third, we excluded patients with non-sinus rhythm to avoid the impact of heart rhythm on renal arterial parameters. The applicability of our findings to patients with non-sinus rhythm remains to be validated.

Fourth, no long term follow up was conducted in this study, which may diminish the overall impact of our findings and deserves future attention. Additionally, the lack of more robust objective measures for evaluating successful sympathetic block in the active group is also a limitation. Further studies should incorporate comprehensive metrics to provide a more objective assessment of sympathetic block efficacy.

Fifth, a methodological limitation of this study is the absence of sham SGB in the control group, which may weaken the design and produce potential subtle biases from the wider care team.

Sixth, although many indicators were discussed, there may still exist missing data related to CSA-AKI, such as comorbidities, postoperative nephrotoxic medications, fluid management, and other subtle management differences among different ICU physicians.

Last, we believe this study should be complemented by animal experiments to measure renal sympathetic nerve activity, assess the expression of related neurotransmitters, and conduct histopathological analysis of renal tissue. These investigations would further elucidate the protective effects of left SGB on perioperative renal function and explore its potential molecular mechanisms. These tests are part of our ongoing research efforts.

## Conclusions

SGB pretreatment can reduce the incidence and severity of CSA-AKI, and promote recovery in patients undergoing cardiac surgery with CPB.

## Data Availability

The datasets used and/or analyzed during the current study are available from the corresponding author upon reasonable request.

## Research registration unique identifying number (UIN)

This study was registered at clinicaltrials.gov (NCT05652179).
